# Comparison of Two Methods for Assaying Reducing Sugars in the Determination of Carbohydrase Activities

**DOI:** 10.1155/2011/283658

**Published:** 2011-05-26

**Authors:** Alexander V. Gusakov, Elena G. Kondratyeva, Arkady P. Sinitsyn

**Affiliations:** ^1^Department of Chemistry, M. V. Lomonosov Moscow State University, Moscow 119991, Russia; ^2^A. N. Bach Institute of Biochemistry, Russian Academy of Sciences, Moscow 119071, Russia

## Abstract

The Nelson-Somogyi (NS) and 3,5-dinitrosalicylic acid (DNS) assays for reducing sugars are widely used in measurements of carbohydrase activities against different polysaccharides. Using twelve commercial enzyme preparations, the comparison of the NS and DNS assays in determination of cellulase, *β*-glucanase, xylanase, and *β*-mannanase activities was carried out. When cellulase activities against CMC were measured, the DNS assay gave activity values, which were typically 40–50% higher than those obtained with the NS assay. In the analysis of the xylanase, *β*-mannanase, and *β*-glucanase activities, the overestimations by the DNS assay were much more pronounced (the observed differences in the activities were 3- to 13-fold). Reasons for preferential use of the NS assay for measuring activities of carbohydrases other than cellulases are discussed.

## 1. Introduction

Carbohydrases (O-glycosidases, or glycoside hydrolases) represent a large class of enzymes hydrolyzing polysaccharides and low-molecular-weight glycosides. They belong to the (EC 3.2.1.−) class of hydrolases. Carbohydrases are classified according to their specificity toward natural glycoside substrates, that is, they are called cellulases, xylanases, mannanases, pectinases, chitinases, and so forth. More recent classification of glycoside hydrolases in families based on amino acid sequence similarities has been proposed by Henrissat [1]. Many carbohydrases found extensive applications in biotechnology [2]. 

Most of the methods for determination of carbohydrase activity are based on the analysis of reducing sugars (RSs) formed as a result of the enzymatic scission of the glycosidic bond between two carbohydrates or between a carbohydrate and a noncarbohydrate moiety. Different methods for assaying the RS have been applied in the carbohydrase activity measurements. The Nelson-Somogyi (NS) assay with copper and arsenomolybdate reagents [3, 4] and the 3,5-dinitrosalicylic acid (DNS) assay described by Miller [5] are the most popular methods used by many researchers. Other methods, such as those based on the use of sodium 2,2^'^-bicinchoninate [6], *p*-hydroxybenzoic acid hydrazide [7], or potassium ferricyanide [8], are less frequently used.

Although the DNS assay is known to be approximately 10 times less sensitive than the NS assay and it does not provide stoichiometric data with oligosaccharides, giving significantly higher values of RS than the actual number of hemiacetal reducing groups [9–11], it has been recommended by the IUPAC commission on biotechnology for measuring standard cellulase activities against filter paper and carboxymethylcellulose (CMC) [12]. The method for determination of xylanase activity with the DNS reagent, reported by Bailey et al. [13], dominates in laboratories throughout the world. The DNS assay has also been used for measuring activities of other carbohydrases, such as amylases, *β*-mannanases, pectinases, and xyloglucanases. [14–19].

The present paper focuses on the comparison of the NS and DNS assays in determination of cellulase, *β*-glucanase, xylanase, and *β*-mannanase activities against different polysaccharides and analysis of the applicability of the assays for correct interpretation of experimental data.

## 2. Materials and Methods

### 2.1. Enzymes and Substrates

Twelve commercial preparations of carbohydrases produced by Adisseo (France), Dyadic International, Inc. (USA), Finnfeeds Finland Oy (Finland), Novozymes (Denmark), and Sibbiofarm (Russia) were used in the studies. CMC (medium viscosity), birchwood glucuronoxylan, and galactomannan (locust bean gum) from Sigma (USA) and barley *β*-glucan and wheat arabinoxylan (both medium viscosity) from Megazyme (Australia) were used as substrates in the enzyme activity measurements.

### 2.2. Enzyme Activity Measurements

The enzymatic hydrolysis of polysaccharides was carried out at pH 5.0 and 50°C in thermostated test tubes (2 mL); in all cases the substrate concentration was 5 mg/mL. The RSs released in hydrolysis were analyzed using the NS [3, 4] and DNS [5] assays modified to smaller volumes (see below). The carbohydrase activities were expressed in international units where one activity unit corresponds to the amount of the enzyme hydrolyzing 1 *μ*moL of glycoside bonds of the substrate per minute. All assays were carried out in duplicates.

**NS Assay**

An aliquot of the substrate stock solution (0.16 mL, 6.25 mg/mL in 0.1 M Na-acetate buffer) was preliminary heated at 50°C for 5 min. Then the enzyme reaction was initiated by adding 0.04 mL of the enzyme solution (also preheated at 50°C for 5 min). The mixture was incubated at 50°C for 10 min (5 min in the case of CMCase and *β*-glucanase activities); the reaction was stopped by addition of 0.2 mL of the Somogyi copper reagent. The tightly stoppered test tube was incubated in a boiling water bath for 40 min; then it was cooled to room temperature and 0.2 mL of the Nelson arsenomolybdate reagent was added. The solution was carefully mixed and incubated for 10 min at room temperature and then 1.4 mL of water was added (0.4 mL of acetone to dissolve the precipitated CMC or *β*-glucan and then 1 mL of water were added in the case of CMCase and *β*-glucanase activity measurements). After centrifugation at 13,000 rpm for 1 min, the absorbance of the supernatant at 610 nm (A_610_) was measured. The A_610_ values for the substrate and enzyme blanks were subtracted from the A_610_ value for the analyzed sample. The substrate and enzyme blanks were prepared in the same way as the analyzed sample except that the necessary amount of the acetate buffer was added to the substrate (enzyme) solution instead of the enzyme (substrate) solution.

**DNS Assay**

An aliquot of the substrate stock solution (0.3 mL, 10 mg/mL in 0.1 M Na-acetate buffer) was mixed with 0.3 mL of the enzyme solution (both solutions were preheated at 50°C for 5 min). After 10 min of incubation at 50°C, 0.9 mL of the DNS reagent was added to the test tube and the mixture was incubated in a boiling water bath for 5 min. After cooling to room temperature, the absorbance of the supernatant at 540 nm was measured. The A_540_ values for the substrate and enzyme blanks were subtracted from the A_540_ value for the analyzed sample. The substrate and enzyme blanks were prepared in the same way as the analyzed sample except that 0.3 mL of the acetate buffer was added to the substrate (enzyme) solution instead of the enzyme (substrate) solution.

## 3. Results and Discussion

Activities of twelve commercial enzyme preparations toward CMC, *β*-glucan, glucuronoxylan, and galactomannan (locust bean gum) are shown in Table 1. When the enzyme activity against CMC was measured, the DNS method gave slightly higher values than the NS method, that is, the ratio of the activities (DNS/NS) was in the range of 1.2–1.7 (data in Table 1 are sorted by this parameter). The average DNS/NS ratio for the CMCase was 1.4 and the standard deviation for the ratio was 0.2. When the enzyme activities against other polysaccharides under study were determined, the observed differences between the DNS and NS assays were much more pronounced. The DNS method gave 3- to 6-fold overestimations of xylanase activity against glucuronoxylan, and the average DNS/NS ratio was 4.0 in this case. When arabinoxylan was used as a substrate in xylanase activity measurements, the difference between the two methods for the RS assay became even more dramatic (data for xylanase activity in Table 1 shown in parentheses), the average DNS/NS ratio being 8.1. The overestimations of similar magnitude for the DNS assay were observed in the *β*-mannanase and *β*-glucanase activity measurements (the average DNS/NS ratios 7.3 and 10.1) with locust bean gum and barley *β*-glucan as substrates. 

The problems associated with the DNS assay have also been reported by other researchers [9–11]. Our data on CMCase activities of twelve enzyme preparations determined with two different RS assays (Table 1) are very similar to those reported by Breuil and Saddler [11], who observed moderate overestimations of the endoglucanase (CMCase) activity of *Trichoderma harzianum *culture filtrates by the DNS assay in comparison with the NS assay. Nevertheless, both methods gave comparable results for the CMCase (activity values of the same decimal magnitude) that should not cause any serious ambiguities if data from different laboratories, obtained with either DNS or NS assay, are analyzed. The reason for higher CMCase activity values obtained with the DNS assay is the fact that cellobiose, being one of the major products produced by cellulases, is decomposed by the DNS itself and is measured as ~1.5 glucoses rather than one reducing end group. So, at least, the use of the DNS assay for measuring standard cellulase activities (either CMCase or filter paper activity [12]) may be justified in the case of cellulase multienzyme preparations deficient by *β*-glucosidase, the enzyme catalyzing the hydrolysis of cellobiose to glucose. In particular, cellulase complexes produced by *Trichoderma *sp. strains are known to be deficient by *β*-glucosidase [20]. In such case the DNS assay may help to obtain more true values of cellulase activity regardless of the *β*-glucosidase level in a cellulase complex. 

The situation with activities of other carbohydrases determined using the DNS assay is more serious. The 3- to 13-fold higher activity values for xylanase, *β*-glucanase, and *β*-mannanase obtained with the DNS assay allow making a conclusion that the comparison of experimental data from various researchers exploiting different RS assays is hardly possible, even if the same substrate is used in activity measurements. Bailey et al. [13] carried out a round robin testing of xylanase activity of a distributed *T. reesei *enzyme preparation against a distributed birchwood glucuronoxylan sample as a substrate. Twenty laboratories participated in this collaborative investigation; thirteen of them used the DNS assay while three laboratories used the NS assay. The activity values obtained with the DNS assay were 3- to 8-fold higher than those obtained with the NS assay. Such DNS/NS ratios are similar to those observed in our studies with a variety of enzyme preparations and glucuronoxylan as a substrate (Table 1). Data on xylanase activity of the distributed enzyme sample, obtained in three laboratories using the variations of the NS assay, were in a very good agreement (the difference did not exceed 20%), while in the laboratories exploiting the DNS assay the up to 3.5-fold activity differences were observed [13]. The 4.2-fold discrepancy in the specific activity of the homogeneous xylanase from *Thermomyces lanuginosus*, determined with the DNS assay by different research teams, may also be mentioned (the reported values of the activity were 212 U/mg [21] and 889 U/mg [22]). However, in spite of the evident drawbacks of the DNS assay, it has been accepted for measuring xylanase activity by most laboratories in the world. 

Other examples of poor applicability of the DNS assay for measuring carbohydrase activity against some polysaccharides may be given. For example, the unusually high side activity of xyloglucanase (Xgh74A) from *Clostridium thermocellum* against barley *β*-glucan (238 U/mg) reported by Zverlov et al. [18] could be attributed to the use of the DNS reagent. According to our data (Table 1), the DNS assay may give 10-fold (or even higher) overestimations when measuring the *β*-glucanase activity. Extremely high specific activity of the already-mentioned Xgh74A against tamarind xyloglucan (295 U/mg) [18] in comparison with other purified xyloglucanases [19, 23] could also be the result of the activity overestimation by the DNS assay. The comparison of HPLC and RS data showed that even milder and less destructive for polysaccharides 2,2^'^-bicinchoninate assay gives some overestimations when applied for measuring xyloglucanase activity [23].

The reason for dramatic overestimations of the carbohydrase activities by the DNS method is the polysaccharide instability under severe conditions of the assay. Most of the known RS assays (including the DNS and NS ones) use boiling of the analyzed sample in alkaline medium. The DNS reagent seems to be the most destructive for polysaccharides, as our (Table 1) and the literature data [9–11] indicate. The polysaccharide degradation under alkaline conditions at high temperature occurs by means of two major mechanisms: hydrolysis and *β*-eliminative depolymerization reaction [24, 25]. For instance, methoxylated pectins are known to be unstable even at room temperature and neutral pH; the *β*-eliminative degradation only takes place at a glycosidic linkage on the nonreducing side of a methoxylated galacturonide residue and this process is dramatically accelerated with increase in pH and temperature [25]. For this reason, both the DNS and NS assay cannot be applied for measuring pectinase activity against pectins (esterified polygalacturonates). Nevertheless, examples of the questionable use of the DNS assay for measuring pectinase activity may be found in the scientific literature [16, 17]. At the same time, polygalacturonic acid, free of the methyl ester groups, can be used as a substrate in determination of the polygalacturonase activity with an RS assay.

## 4. Conclusions

In summary, when cellulase activities against CMC are measured, the DNS assay gives activity values of similar magnitude as those obtained by the NS assay, the first method typically providing 40–50% higher numbers. In this case, the interpretation of experimental data obtained by both methods should not cause ambiguities. However, the DNS assay gives 3- to 6-fold overestimations of xylanase activity against glucuronoxylan compared to the NS assay. When the *β*-glucanase, *β*-mannanase, or xylanase activity against arabinoxylan is measured, the overestimations by the DNS assay become more pronounced (up to 13-fold). So the comparison of data reported from different laboratories should be taken with care. Both methods cannot be applied for measuring pectinase activity against methylated pectins.

## Figures and Tables

**Table 1:** Activities (U/mL or U/g) of different enzyme preparations toward CMC, barley *β*-glucan, birchwood glucuronoxylan, and galactomannan measured using the DNS and NS assays.

No.	CMCase			*β*-Glucanase			Xylanase			*β*-Mannanase		
	DNS	NS	DNS/NS	DNS	NS	DNS/NS	DNS	NS	DNS/NS*	DNS	NS	DNS/NS
(1)	131	112	1.2	5049	544	9.3	1215	353	3.4 (5.5)	329	36	9.1
(2)	2821	2382	1.2	35624	3051	11.7	8381	2867	2.9 (5.7)	—	—	—
(3)	119	86	1.4	673	79	8.5	345	88	3.9 (8.0)	118	16	7.4
(4)	374	267	1.4	3275	288	11.4	1380	490	2.8 (11.5)	359	40	9.0
(5)	5995	4246	1.4	31915	2628	12.1	3933	1173	3.4 (8.4)	—	—	—
(6)	71	50	1.4	2171	224	9.7	1029	173	5.9 (8.8)	202	33	6.1
(7)	124	83	1.5	692	117	5.9	1694	500	3.4 (5.8)	96	11	8.7
(8)	127	85	1.5	11770	916	12.8	1890	507	3.7 (5.8)	386	76	5.1
(9)	63	41	1.5	636	62	10.3	1379	283	4.9 (8.5)	106	15	7.1
(10)	11755	7607	1.5	71920	6284	11.4	74350	24417	3.0 (11.2)	—	—	—
(11)	9474	5876	1.6	60611	5806	10.4	321904	58600	5.5 (10.4)	982	157	6.3
(12)	132	79	1.7	3591	469	7.7	908	161	5.6 (8.1)	1040	145	7.2

Average ratio			1.4			10.1			4.0 (8.1)			7.3

Std. deviation			0.2			2.0			1.1 (2.1)			1.4

*Ratio of xylanase activities determined with arabinoxylan from wheat as a substrate is given in parentheses.
